# Effect of Self-Efficacy on Bedtime Procrastination Among Chinese University Students: A Moderation and Mediation Model

**DOI:** 10.3389/fpsyg.2022.863523

**Published:** 2022-05-16

**Authors:** Xiaolu Meng, Haodong Su, Chunlu Li

**Affiliations:** ^1^Department of Psychology, School of Medical Humanitarians, Guizhou Medical University, Guiyang, China; ^2^School of Humanities and Social Sciences, Binzhou Medical University, Yantai, China

**Keywords:** self-efficacy, bedtime procrastination, harm avoidance, novelty seeking, tridimensional personality questionnaire

## Abstract

Bedtime procrastination (BP) is generally considered to be a maladaptive behavior. However, BP may be an adaptive fast LH strategy within the LH framework, and further, personal beliefs about their abilities and resources promote this fast LH strategy. Here, the present study addressed this idea, focusing on the effect of self-efficacy on BP, the mediation of harm avoidance (HA), and the moderation of novelty seeking (NS). Data from 552 Chinese university students (205 men and 347 women) were analyzed using SPSS 25.0 and SPSS PROCESS Macro. Results indicated that HA partially mediates the relationship between self-efficacy and BP. Main interactional effects have been observed when NS is introduced in the model as a moderator. Implications and limitations of the study and suggestions for further study are discussed.

## Introduction

Sleep serves important functions and is essential for physical and mental health (Banks and Dinges, 2007). Insufficient sleep leads to a lot of adverse consequences, such as low working efficiency (Kessler et al., 2011), poor academic performance (Jiang et al., 2011), a reduction in optimism and sociability (Haack and Mullington, 2005; Lemola et al., 2011), mental stress, depressed mood, and anxiety (Da Costa et al., 2010). An important behavioral factor held responsible for insufficient sleep in the general population is bedtime procrastination (BP), a phenomenon defined as “going to bed later than intended, without having external reasons for doing so” (Kroese et al., 2014, 2016). BP was found to be positively correlated with short sleep duration and poor sleep quality among Chinese (Zhang and Wu, 2020) and Polish (Herzog-Krzywoszanska and Krzywoszanski, 2019) university students. Further, university students have higher BP scores than non-student groups (Herzog-Krzywoszanska and Krzywoszanski, 2019). However, the psychological mechanisms of BP are still not fully understood. BP is well-studied from a self-regulation perspective. For example, BP is supposed to be a self-regulation problem with a poor ability to resist temptations (Kroese et al., 2016). Consistent with this perspective, BP has been found to be negatively associated with self-regulation (Kroese et al., 2014, 2016) and positively related to the level of depleted self-regulatory resources (Kamphorst et al., 2018). Further, people who think willpower is limited and easily depleted are more likely to procrastinate at bedtime following a stressful day, but not less stressful days (Bernecker and Job, 2020). That is to say, a lack of self-regulation skills or psychological resources leads to a failure of self-regulation, which in turn leads to BP. In this perspective, BP is maladaptive because it will lead to insufficient sleep which in turn results in the aforementioned adverse consequences in the long term. However, the evolutionary origin and function of BP within the life history (LH) framework has not been investigated, and BP might be adaptive within the LH framework.

Life history theory has been developed to explain differences in energy and time allocation patterns between and within species (Sng et al., 2017). All living organisms have limited resources. How they allocate their limited resources is critical to the survival and continuation of species. Their resource allocation strategies often change based on an assessment of the environmental constraints. In a predictable living environment, it is cost-effective to plan and work for higher future rewards. Therefore, humans and animals' cognition and behavior are biased toward the slow LH strategy and their cognition and behavior tend to be more future- than present-oriented. This is to say, they prefer the behaviors that are likely to have high returns in the future but have little or no immediate benefit. Within the same framework, people with slow LH are expected to be future-oriented and not procrastinate (Chen and Chang, 2016). On the contrary, when the future is uncertain and less predictable, there is a low probability that the investment will pay off in the future. Therefore, a fast strategy is more adaptive, wherein organisms will show an increased focus on the present and discount the future. With the same logic, fast LH people are expected to be procrastinators because investing in the present is the most profitable compared with the future (Chen and Chang, 2016).

The same logic seems to also apply to BP. For example, a recent study found that people who believed that willpower was limited and easily depleted (limited theory) were more likely to procrastinate their bedtime after a stressful day than those who considered willpower as a non-limited resource (non-limited theory), whereas there was no difference between them after a less stressful day (Bernecker and Job, 2020). The author argued that since sleep may be the best way to recover, people with a limited vs. non-limited theory should be more concerned with restoring their resources and going to bed on time after a stressful day. However, they ironically procrastinated more at bedtime. Within the LH framework, it seems that BP is a fast LH strategy for people with a limited theory after a stressful day, who prefer immediate relaxation to long-term benefits. People with a limited theory believe that their willpower resources are easily depleted and that they need to be restored, for instance, by taking a break or eating, to be available again. Besides, people with a limited vs. non-limited theory are expected to be more exhausted from unpleasant tasks following a demanding day (Bernecker and Job, 2015). BP serves the adaptive function of taking an immediate break from the challenge and avoiding serious negative consequences and death from exhaustion when there is no more psychological resource for ongoing and upcoming challenges.

On the other hand, the results of this study also suggested that personal beliefs about their abilities and resources promote the fast LH strategy. Since these beliefs can influence their assessment of the controllability of the environment. For example, unpredictability schemas in college students are associated with lower self-efficacy (Ross et al., 2016), the belief in one's competence to cope with a broad range of stressful or challenging demands (Bandura, 1986). Further, people with a fast LH strategy have a lower self-efficacy score than slow LH strategy individuals when they are consumers (Hidding and Fennis, 2018). Therefore, low self-efficacy is expected to promote the fast LH strategy, and self-efficacy might be negatively correlated with BP (H1).

In a stressful situation, low self-efficacy increases subjective assessments of environmental unpredictability (Ross et al., 2016). Fear of such unpredictability will lead individuals to over-prepare for or escape from such situations. However, at the same time, due to low self-efficacy, the need for over-preparation makes individuals face greater challenges and exhaustion, which in turn further increases the subjective experience of unpredictability and uncontrollability. In this context, escaping from stressful situations and immediately letting oneself relax becomes the best option at that moment. Therefore, fear and avoidance of environmental unpredictability are expected to mediate the effect of self-efficacy on BP. The harm avoidance (HA) in the tridimensional personality questionnaire (TPQ) paints a good picture of such fear and avoidance tendencies by four facets: HA1 (anticipatory worry), HA2 (fear of uncertainty), HA3 (shyness with strangers), and HA4 (fatigability and asthenia). More importantly, HA is especially associated with serotonin (Cloninger, 1986), an important neurotransmitter involved in negative emotions, such as depression and anxiety. Further, convergent lines of evidence suggest a negative correlation between self-efficacy and negative affect in undergraduate students (Ashby and Kottman, 2000; Leganger et al., 2000) and Chinese adolescents (Huang and Zhang, 2010), while, negative affect was positively associated with a fast LH strategy (Figueredo et al., 2007; Sefcek, 2007). In addition, a recent study found a positive relation between BP and negative affect, and the effect of self-compassion on reducing BP was mediated by lower negative affect but not higher positive affect (Sirois et al., 2019). Based on these data, we assume that HA mediates the effects of self-efficacy on BP (H2).

Chinese college students are usually undergoing a learning and growth period that is completely different from high school. In high school, they are exposed to the strict disciplines or supervision imposed by family members and teachers; while these supervisions are no longer in the college, making self-discipline crucial for them (Geng et al., 2018). This is the first time that they have faced personal and academic challenges independently. On the other hand, they are relatively inexperienced in independence and have not yet reached psychosocial maturity, especially the ability to restrain themselves in the face of emotional, exciting, or risky stimuli (Icenogle et al., 2019). Therefore, the next question was whether the relationship between self-efficacy and BP might be moderated by poor self-discipline processes facing emotional, exciting, or risky stimuli, such as novelty seeking (NS). NS in TPQ refers to a tendency to respond to novel stimuli with excitement. It strongly resembles sensation seeking (Zuckerman and Cloninger, 1996). Several studies revealed that sensation seeking was positively associated with later bedtime preference, such as eveningness (sometimes labeled “owls”) in young adults (Tonetti et al., 2010; (Antúnez et al., 2014; Geng et al., 2018)). Further, NS is strongly linked with the defect in the probability and delay discounting task (Zheng et al., 2019), which is related to the present-oriented behavior and cognition of the fast LH strategy. Therefore, we hypothesize that NS moderates both the direct effect of self-efficacy on the BP (H3) and the indirect effect of self-efficacy on the BP (H4), which is mediated by HA.

**Figure 1:**
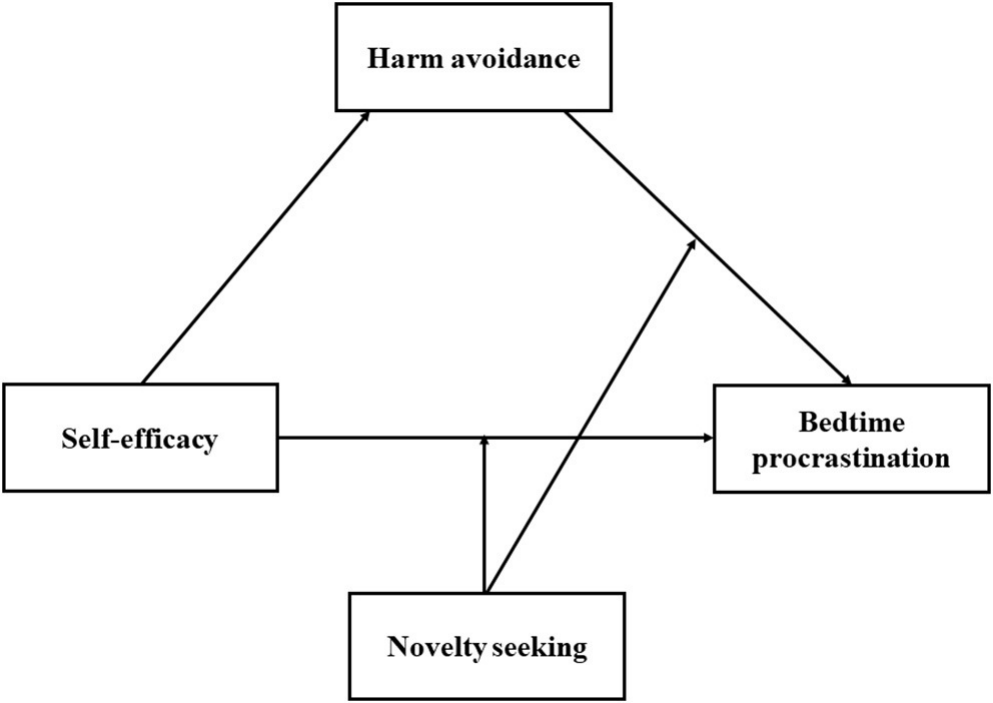
Hypothesized moderated mediation model to predict bedtime procrastination.

Together, we aim to examine these four following hypotheses (as shown in Figure 1):

H1: Self-efficacy is negatively related to BP.

H2: HA mediates the effect of self-efficacy on BP.

H3: NS moderates the direct effect of self-efficacy on the BP.

H4: NS moderates the indirect effect of self-efficacy on the BP mediated by HA.

## Materials and Methods

### Subjects

This research was approved by the ethics committee of Guizhou Medical University. The data were collected through Wen Juan Xing, an online survey tool. In total, 600 Chinese college student volunteers were recruited from five universities located in Guizhou, Jiangxi, Henan, Shandong Province, and Shanghai, respectively. An anonymous self-report questionnaire was distributed to volunteers during their elective courses. All participants gave informed consent and had about 20 min to complete every questionnaire item. To ensure the quality of the data, we excluded the data of subjects whose completion time was <300 s. Finally, data from 552 participants (205 men and 347 women) with an age range of 18–23 years (M = 19.22 years, SD = 0.643 years) were included in the analysis.

### Measures

#### BP Scale (BPS)

Bedtime procrastination was assessed by a Chinese version of the 9-item BP scale (Xiao-han et al., 2021) where items were scored on a 5-Likert point scale, 1 = almost never, 5 = almost always. The Cronbach's coefficient in this study was 0.831.

#### The General Self-Efficacy Scale (GSES)

General self-efficacy (GSE) was measured by a 10-item general self-efficacy scale (GSES) designed to assess optimistic self-beliefs to cope with a variety of difficult demands in life (Schwarzer and Jerusalem, 1995). The Chinese version of the GSES was used (Cai-kang et al., 2001). Items were scored on a 5-Likert point scale, 1 = almost never, 5 = almost always. Cronbach's alpha in the present sample was 0.877.

#### Tridimensional Personality Questionnaire (TPQ)

The NS and HA were evaluated by a Chinese version of the 100-item TPQ, which is a true/false questionnaire (Cai-kang et al., 2001). The NS dimension is composed of four facets: NS1 (exploratory excitability), NS2 (impulsivity), NS3 (extravagance), and NS4 (disorderliness). The HA dimension includes four facets: HA1 (anticipatory worry), HA2 (fear of uncertainty), HA3 (shyness with strangers), and HA4 (fatigability and asthenia), while the RD also has four facets: RD1 (sentimentality), RD2 (persistence), RD3 (attachment), and RD4 (dependence). The Cronbach's coefficient in the current study was 0.901.

### Data Analysis

Data collected in this study were processed using SPSS 25.0. Following initial correlation analysis, we used SPSS PROCESS Macro Model 4 to examine whether HA mediates the association between self-efficacy and BP (H2). Bootstrapped confidence interval (*CI*) (5,000 bootstrap samples) for the indirect effect was obtained. Then, we used model 15 of the PROCESS to assess the moderated mediation model (H3 and H4, as shown in Figure 1).

## Results

### The Descriptive Statistics and Correlations for the Variables

Table 1 presents the descriptive statistics and correlations for the variables in the present study. Self-efficacy correlated strongly with HA (*r* = −0.43, *p* < 0.01) and BP (*r* = −0.33, *p* < 0.01), and did not relate with NS (*r* = −0.08, *p* > 0.05). HA was positively correlated with BP (*r* = 0.22, *p* < 0.01) and did not relate with NS (*r* = −0.07, *p* > 0.05). NS was positively correlated with BP (*r* = 0.29, *p* < 0.01).

**Table 1:** Descriptive statistics and correlations of study variables.

**Variables**	**M**	**SD**	**1**	**2**	**3**	**4**
1. Self-efficacy	32.92	5.37	–			
2. Harm avoidance	51.60	5.90	−0.43**	–		
3. Novelty seeking	46.46	4.21	−0.08	−0.07	–	
4. Bedtime procrastination	29.27	6.97	−0.33**	0.22**	0.29**	–

N = 552, **p < 0.01.

### Hypothesis Test

We expected that self-efficacy is negatively related to the BP and that HA would mediate (H2), while NS moderate the direct association between self-efficacy and BP (H3) and the indirect effect of self-efficacy on the BP mediated by HA (H4). First, to test the mediation hypothesis (H2), model 4 of PROCESS macro for SPSS (Hayes, 2017) was used. The specifications of this model can be seen in Table 2. Results show that self-efficacy is negatively and significantly related to HA (*R*^2^ = 0.19; *p* < 0.001). Self-efficacy is negatively and significantly related with BP (*R*^2^ = 0.11; *p* < 0.001), which supports H1. After controlling for gender, grade and age, the mediator and dependent variable models show that self-efficacy negatively predicted HA (β = −0.48, *p* < 0.001), HA positively predicted BP (β = 0.12, *p* < 0.05), and self-efficacy negatively predicted BP (β = −0.36, *p* < 0.001). The resampling procedure (5,000 bootstrap samples) indicates a significant indirect effect since the *CI* at 95% does not include the value of zero (as shown in Table 2). These results indicated a significant mediating effect of HA in the relationship between self-efficacy and BP. H2 is confirmed. Our mediation model explains 14.3% of the BP.

**Table 2:** Mediation analysis for self-efficacy, harm avoidance (HA), and bedtime procrastination (BP).

	**β**	**SE**	**t**	**p**
Mediator variable model				
Constant	57.20***	10.89	5.25	<0.001
Gender	0.21	0.48	0.43	0.67
Age	0.53	0.58	0.92	0.36
Grade	−0.23	0.52	−0.44	0.66
Self-efficacy	−0.48***	0.04	−11.06	<0.001
Dependent variable model				
Constant	19.18	13.59	1.41	0.16
Gender	0.43	0.59	0.73	0.47
Age	0.83	0.71	1.18	0.24
Grade	−0.55	0.64	−0.86	0.39
Self-efficacy	−0.36***	0.06	−6.12	<0.001
Harm avoidance	0.12*	0.05	2.21	<0.05
				
	**β**	**Boot SE**	**BootLLCI**	**BootULCI**
Total effect	−0.42	0.05	−0.52	−0.31
Direct effect	−0.36	0.06	−0.48	−0.24
Indirect effect	−0.06	0.02	−0.10	−0.01

N = 552. LL, low limit; CI, confidence interval; UL, upper limit. Gender was dummy coded. Bootstrap sample size = 5,000. *p < 0.05 and ***p < 0.001.

**Figure 2:**
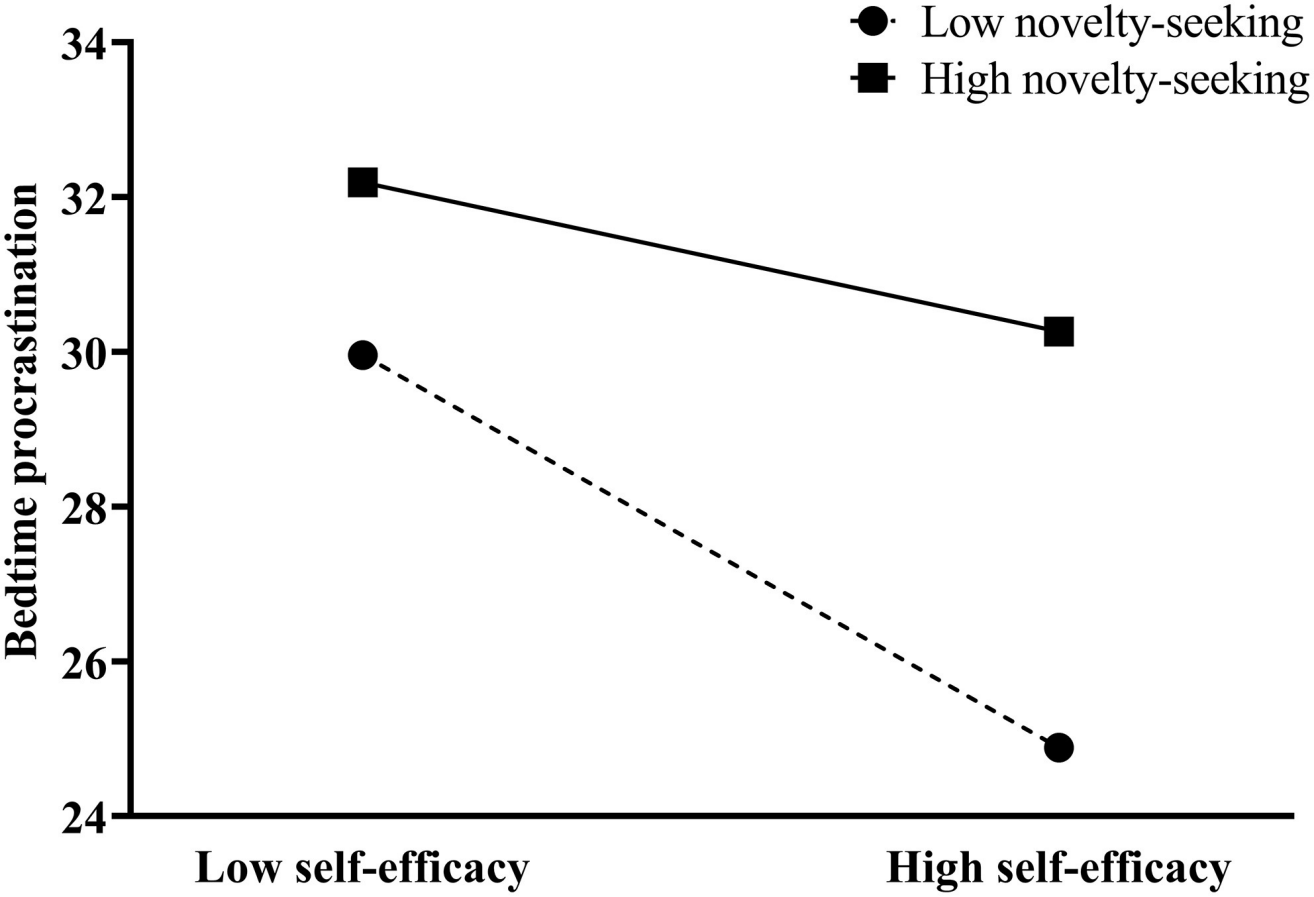
The moderation effect of novelty seeking on self-efficacy to bedtime procrastination.

**Figure 3:**
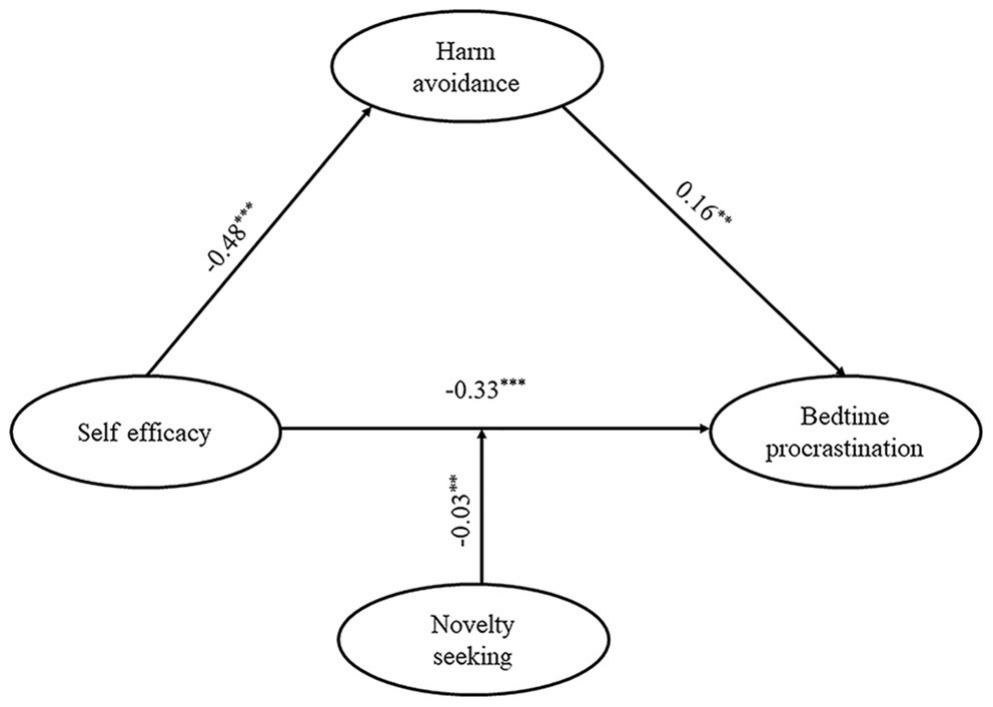
Theoretical research model with standard coefficients.

To test H3 and H4, we performed model 15 of the PROCESS macro for SPSS (Hayes, 2017). The specification of this model can be seen in Table 3. The results showed that the interaction effect of HA and NS on BP was insignificant (β = −0.01, *p* = 0.41, as shown in Table 3). Therefore, H4 was not supported. That is to say, the magnitude of the indirect effect of self-efficacy on the BP mediated by HA did not change according to NS. The results, on the other hand, revealed a significant interaction effect of self-efficacy and NS on BP (β = 0.03, *p* < 0.05). As can be seen from the conditional direct effect analysis, three conditional direct effects were negatively and significantly different from zero. Thus, H3 was supported. Namely, the effect of self-efficacy on BP changed according to NS (as shown in Figure 2). The final model is shown in the Figure 3.

**Table 3:** Moderated mediation analysis for self-efficacy, HA, BP, and novelty seeking (NS).

	**β**	**SE**	**t**	**p**
Mediator variable model				
Constant	−10.30	10.73	−0.96	0.34
Gender	0.21	0.48	0.43	0.67
Age	0.53	0.58	0.92	0.36
Grade	−0.23	0.52	−0.44	0.66
Self-efficacy	−0.48***	0.04	−11.06	<0.001
Dependent variable model				
Constant	12.90	12.44	1.04	0.30
Gender	0.24	0.56	0.42	0.67
Age	0.88	0.67	1.31	0.19
Grade	−0.72	0.61	−1.19	0.24
Self-efficacy	−0.33***	0.06	−5.78	<0.001
Harm avoidance	0.16**	0.05	3.25	<0.01
Novelty seeking	0.46***	0.06	7.09	<0.001
Self-efficacy × Novelty seeking	0.03*	0.01	2.20	<0.05
Harm avoidance × Novelty seeking	−0.01	0.01	−0.83	0.41
				
	**β**	**Boot SE**	**BootLLCI**	**BootULCI**
Conditional direct effect analysis				
M – 1SD (−4.20)	−0.45	0.08	−0.62	−0.29
M (0.00)	−0.33	0.06	−0.44	−0.22
M + 1SD (4.20)	−0.20	0.08	−0.35	−0.05
Conditional indirect effect analysis				
M – 1SD (−4.20)	−0.10	0.04	−0.17	−0.02
M (0.00)	−0.08	0.03	−0.13	−0.03
M + 1SD (4.20)	−0.06	0.03	−0.12	0.01

N = 552. LL, low limit; CI, confidence interval; UL, upper limit. Gender was dummy coded. Bootstrap sample size = 5,000. *p < 0.05, **p < 0.01, and ***p < 0.001.

## Discussion

Bedtime procrastination, an important behavioral factor responsible for insufficient sleep in the general population, is an emerging field of procrastination research in recent years (Kroese et al., 2014, 2016). Although various causal factors of BP research have been investigated, the mechanisms underlying BP appear to be more complex than expected and less clear than other forms of procrastination. BP is generally considered to be a maladaptive behavior. However, a recent study found that stress has a moderate role in the relationship between BP and beliefs about willpower (Bernecker and Job, 2020), suggesting that BP might be a fast LH strategy and serve an adaptive function within the LH framework, and that personal beliefs about their abilities and resources promote this fast LH strategy. Here, we further addressed this idea, focusing on the effect of the self-efficacy on BP, the mediation of HA, and the moderation of NS.

First, we confirm that self-efficacy is an important factor negatively and significantly related with BP. This is in line with previous studies showing a negative correlation between self-efficacy and procrastination (Steel, 2007; Wäschle et al., 2014) and sleep problems (Przepiórka et al., 2019).

Second, we identified a partially mediated role of negative affect on the relationship between self-efficacy and BP. The less self-efficacy, the more negative affect (HA) and finally results in more BP. This was consistent with the result of previous research, where a positive relation between BP and negative affect was found, and the effect of self-compassion on reducing BP was mediated by lower negative affect but not higher positive affect (Sirois et al., 2019). According to LH theory, the negative effect might promote the fast LH strategy. Sleep appears to be the best way to recover, however, its restorative effects are not experienced until the next morning, while the relaxing effect of leisure and social activities before going to bed (Chung et al., 2020) can be experienced immediately. Thus, prolong relaxing activities into the night might be the best recovery way for bedtime procrastinator within the LH framework.

Finally, this study explores the facilitating effect of the direct association between self-efficacy and BP and the indirect effect of self-efficacy on the BP mediated by HA. Our results showed that NS did not moderate the indirect effect of self-efficacy on the BP mediated by HA. On the other hand, a significant interaction between self-efficacy and NS was found. High novelty seekers have high BP regardless of self-efficacy; while for people with low NS, lower self-efficacy was associated with higher BP. These results suggest that it might be a potentially effective intervention to improve the self-efficacy for the bedtime procrastinators with low NS, but not for those with high NS.

This study makes several contributions. At a theoretical level, it improves our understanding of the mechanisms of BP. It might be an active adaptive strategy within the LH framework. The stress-related personality traits, such as self-efficacy, the HA, and NS, interact with each other to influence BP. At a practical level, this study shows that negative affect, such as HA, might be a target of BP intervention; besides, clients' novel seeking types need to be considered when formulating self-efficacy interventions for BP.

The present study had some limitations. First, it was a cross-sectional design which does not allow establishing the causality of mediation (Zapf et al., 1996). Second, we collected a low number of sociodemographic variables, which limits the possibility to explore how the moderation and mediation model works with different groups of people. We only collected three demographic variables, such as age, gender, and grade, limiting the possibility to explore whether the mediation model work with different groups.

## Conclusion

A lower sense of self-efficacy in dealing with external stressful events leads to BP. HA mediated and NS moderated the effect of self-efficacy on the BP.

## Data Availability Statement

The original contributions presented in the study are included in the article, further inquiries can be directed to the corresponding author.

## Ethics Statement

The studies involving human participants were reviewed and approved by the Ethics Committee of Guizhou Medical University. The participants provided their written informed consent to participate in this study.

## Author Contributions

XM and CL designed and performed the research. XM, CL, and HS analyzed the data. CL wrote the manuscript. XM, HS, and CL revised the manuscript. All authors contributed to the article and approved the submitted version.

## Funding

This research was funded by the Guizhou Medical University start-up fund for doctoral talent (J-[2021]050; J-[2021]051).

## Conflict of Interest

The authors declare that the research was conducted in the absence of any commercial or financial relationships that could be construed as a potential conflict of interest.

## Publisher's Note

All claims expressed in this article are solely those of the authors and do not necessarily represent those of their affiliated organizations, or those of the publisher, the editors and the reviewers. Any product that may be evaluated in this article, or claim that may be made by its manufacturer, is not guaranteed or endorsed by the publisher.
